# The spike receptor-binding motif G496S substitution determines the replication fitness of SARS-CoV-2 Omicron sublineage

**DOI:** 10.1080/22221751.2022.2111977

**Published:** 2022-08-31

**Authors:** Ronghui Liang, Zi-Wei Ye, Chon Phin Ong, Zhenzhi Qin, Yubin Xie, Yilan Fan, Kaiming Tang, Vincent Kwok-Man Poon, Chris Chung-Sing Chan, Xiaomeng Yang, Hehe Cao, Kun Wang, Haoran Sun, Bodan Hu, Jian-Piao Cai, Cuiting Luo, Kenn Ka-Heng Chik, Hin Chu, Yi Zheng, Kwok-Yung Yuen, Jasper Fuk-Woo Chan, Dong-Yan Jin, Shuofeng Yuan

**Affiliations:** a State Key Laboratory of Emerging Infectious Diseases, Carol Yu Centre for Infection, Department of Microbiology, School of Clinical Medicine, Li Ka Shing Faculty of Medicine, The University of Hong Kong, Pokfulam, Hong Kong Special Administrative Region, People’s Republic of China; b School of Biomedical Sciences, Li Ka Shing Faculty of Medicine, The University of Hong Kong, Pokfulam, Hong Kong Special Administrative Region, People’s Republic of China; c Centre for Virology, Vaccinology and Therapeutics, Hong Kong Science and Technology Park, Shatin, Hong Kong Special Administrative Region, People’s Republic of China; d Department of Infectious Disease and Microbiology, The University of Hong Kong-Shenzhen Hospital, Shenzhen, Guangdong Province, People’s Republic of China; e Key Laboratory of Infection and Immunity of Shandong Province, Department of Immunology, School of Basic Medical Sciences, Cheeloo College of Medicine, Shandong University, Jinan, People’s Republic of China; f Department of Microbiology, Queen Mary Hospital, Pokfulam, Hong Kong Special Administrative Region, People’s Republic of China; g Academician Workstation of Hainan Province, Hainan Medical University-The University of Hong Kong Joint Laboratory of Tropical Infectious Diseases, Hainan Medical University, Haikou, People’s Republic of China; h Guangzhou Laboratory, Guangdong Province, People’s Republic of China

**Keywords:** COVID-19, Omicron BA.1, Omicron BA.2, fitness, G496S

## Abstract

The replication and pathogenicity of SARS-CoV-2 Omicron BA.2 are comparable to that of BA.1 in experimental animal models. However, BA.2 has rapidly emerged to overtake BA.1 to become the predominant circulating SARS-CoV-2 variant worldwide. Here, we compared the replication fitness of BA.1 and BA.2 in cell culture and in the Syrian hamster model of COVID-19. Using a reverse genetics approach, we found that the BA.1-specific spike mutation G496S compromises its replication fitness, which may contribute to BA.1 being outcompeted by BA.2 in the real world. Additionally, the BA.1-unique G496S substitution confers differentiated sensitivity to therapeutic monoclonal antibodies, which partially recapitulates the immunoevasive phenotype of BA.1 and BA.2. In summary, our study identified G496S as an important determinant during the evolutionary trajectory of SARS-CoV-2.

## Introduction

The Coronavirus Disease 2019 (COVID-19) pandemic has led to over 5.6 million deaths globally since first reported in late 2019 [1,2]. Despite population-wide vaccination campaigns in many countries, severe acute respiratory syndrome coronavirus 2 (SARS-CoV-2) continues to disseminate globally more than 2 years since the pandemic started due to the high prevalence of reinfection and vaccine-breakthrough infections among individuals with waning neutralizing antibody titres [3]. The persistence of SARS-CoV-2 in the human population is also contributed by the appearance of variants, especially those designated as Variants of Concern (VOCs) by the World Health Organization (WHO). VOCs are known to be highly transmissible and able to partially evade immunity induced by natural infection or vaccination. The Alpha (B.1.1.7) variant emerged in mid-2020 and quickly outcompeted the Beta (B.1.351) variant [4]. The Delta (B.1.617.2) variant with enhanced transmissibility and moderate level of antibody resistance then replaced the Alpha (B.1.1.7) variant since mid-2021 [5].

The recently emerged Omicron variant, first found in South Africa, Botswana, and Hong Kong in November 2021, has spread at an unprecedented speed, with a doubling time of only 2–3 days [6,7]. The Omicron variant of severe acute respiratory syndrome coronavirus 2 (SARS-CoV-2) emerged in November 2021 and rapidly replaced the Delta variant as the predominant circulating variant because of its higher transmissibility and immune evasiveness. Among the major lineages or subvariants of Omicron, BA.1 (B.1.1.529.1) and BA.2 (B.1.1.529.2) have been the most intensely studied. In Syrian hamsters and K18-human angiotensin converting enzyme 2 (hACE2)-transgenic mice, BA.1 and BA.2 are comparably pathogenic and replicative, but cause less severe disease than wild-type and previous SARS-CoV-2 variants [8]. In terms of immunoevasion to antibodies elicited by vaccination or natural infection, BA.1 and BA.2 are comparable with each other [3]. However, the comparative intrinsic fitness of the two subvariants, which may help to explain why BA.2 has overtaken BA.1 to become the predominant subvariant in different countries (https://covid.cdc.gov/covid-data-tracker/#variant-proportions), remains unclear. The spike protein of BA.1 contains 17 amino acid substitutions, 3 deletions, and 1 insertion that are different from that of BA.2 [9]. Many of these amino acid mutations are found in the N-terminal domain (NTD) and receptor-binding domain (RBD) of the spike proteins and may affect the virus’ fitness. In this study, we investigated the comparative fitness of BA.1 and BA.2, and found that BA.2 exhibited a significant fitness advantage over BA.1 *in vitro* and *in vivo*. Using a reverse genetic approach, we found that the BA.1-specific spike mutation G496S reduces its replication fitness, which may contribute to the replication advantage of BA.2 to BA.1. Intriguingly, the BA.1-unique G496S substitution exhibited different sensitivity to therapeutic monoclonal antibodies, which may contribute to the different antibody evasion properties of Omicron sublineages [10,11]. Importantly, our study identified G496S as an important determinant during the evolutionary trajectory of SARS-CoV-2.

## Materials and methods

### Viruses and cells

SARS-CoV-2 Omicron BA.1 and BA.2 variants were isolated from respiratory tract specimens of laboratory confirmed COVID-19 patients in Hong Kong by RT–PCR and genome sequencing. The virus-inoculated VeroE6-TMPRSS2 cells were monitored daily for cytopathic effects by light microscopy and the cell supernatants were collected daily for qRT-PCR to assess viral load. The viruses were passaged two times in VeroE6-TMPRSS2 cells before being used for the experiments. Nanopore sequencing was performed to confirm a lack of changes before and after virus passage. The sequences have been deposited in GISAID with the following accession codes: Omicron BA.1: EPI_ISL_7385702 [12]; Omicron BA.2: EPI_ISL_9845731 [13]. VeroE6-TMPRSS2 cells were maintained in DMEM culture medium supplemented with 10% heat-inactivated FBS, 50 U/ml of penicillin and 50 μg/ml of streptomycin. All experiments involving live SARS-CoV-2 followed the approved standard operating procedures of the Biosafety Level 3 facility at The University of Hong Kong [14].

### *In vitro* virus competition assay

Approximately 1 × 10^5^ cells were seeded onto each well of 24-well plates and cultured at 37°C, 5% CO_2_ for 24 h. Equal PFUs of two variants (1:1 ratio) were inoculated onto VeroE6-TMPRSS2 and Caco2 cells at a final MOI of 0.10 for each variant. The mixed viruses were incubated with the cells at 37°C for 2 h. After infection, the cells were washed twice with PBS to remove residual viruses. One millilitre of culture medium was added into each well. At each time-point, 350 µl of cell culture lysate and/or supernatant was collected for RNA extraction. Ratios of variant’s RNA were determined via RT–PCR with quantification of Sanger peak heights. All samples were stored at minus 80°C until analysis.

### *In vivo* virus competition models

To evaluate the competitive fitness of different variants *in vivo*, virus competition experiments were performed in the established golden Syrian hamster model of COVID-19 as previously described [12]. The animal experiments were approved by the Institutional Review Board of The University of Hong Kong Committee on the Use of Live Animals in Teaching and Research (CULATR). Eight to ten weeks old male Syrian hamsters were obtained from the Chinese University of Hong Kong Laboratory Animal Service Centre through the HKU Centre for Comparative Medicine Research. The hamsters were maintained in Biosafety Level 2 housing and fed with standard pellet feed and water *ad libitum*. The animals were infected each intranasally with 10^5^ PFU of a mixture of both viruses (1:1 ratio). At indicated time-point, the hamsters were sacrificed for sampling. Total RNA was extracted from the nasal turbinate, trachea, and lung tissues of the hamsters at indicated time-point using RNeasy kit (Qiagen) for viral load quantification and calculation of the relative viral-load ratio.

### Validation of competition assay

The experiments were performed as previously described [15]. To validate the consistency and accuracy of the competition assay, the indicated variants were mixed at ratios of 10:1, 5:1, 3:1, 1:1, 1:3, 1:5, and 1:10 based on their PFU titres (total 10^5^ PFU viruses). The total RNA of these mixed variants was isolated and amplified by RT–PCR followed by Sanger sequencing. The variant’s ratio was calculated by the peak heights of Sanger sequencing. Data were analysed by linear regression with correlation coefficients (*r*) and significance (*P*).

### Quantification of variant-to-variant ratios

Sanger sequencing and/or next-generation sequencing (NGS) were utilized to reliably quantify the relative amounts of variant in mixed specimens. For Sanger sequencing, RT–PCR was conducted using a SuperScript™ III One-Step RT–PCR kit (Invitrogen, Carlsbad, CA, USA) to amplify the extracted viral RNA (Qiagen). The primers used are listed in Supplementary Table 1. Relative replicative fitness values for variant_A compared to variant_B were analysed according to *w = *(*f0/i0*), where *i0* was the initial variant_A /variant_B ratio and *f0* was the final variant_A/variant_B ratio after competition. Sanger sequencing (initial time-point T0) counts for each variant being compared were based upon average counts over repeated samples of inoculum per experiment, and post-infection (time-point T1) counts were taken from samples of individual subjects. To model *f0/i0*, the ratio T0/T1 was determined for each subject in distinct strain groups. The NGS was done by Novogene Bioinformatics Technology Co., Ltd (Tianjian, China) with the NovaSeq System, Paired-end 250 (PE250). After quality control, the cDNA was randomly sheared into short fragments. The obtained fragments were end repaired, A-tailed and further ligated with Illumina adapter. The fragments with adapters were PCR amplified, size selected, and purified. High-throughout sequencing of those clusters was performed on the platform of NovaSeq 6000 with the PE250 read length. The output fastq files were subjected to adapter removal using FASTP. The pair end reads were then combined into one read using Paired-End reAd merger (PEAR). Finally, the annotation section was performed with BWA software, followed by calling variation with GATK. The ratios between the comparative variants were calculated based on the ratio of reads containing the two spike mutations, G496S and G498R.

### Quantification of viral yield of omicron BA.1 and BA.2 variants

Quantitative reverse transcription-polymerase chain reaction (qRT-PCR) was utilized to measure the viral load of samples, while 50% tissue culture infectious dose (TCID_50_) assay was employed for live virus titration. For qRT-PCR, the QuantiNova RT–PCR kit (Qiagen) was used with a LightCycler 480 Real-Time PCR System (Roche) as previously described [16]. The primers used are listed in Supplementary Table 1.

### Interactive structure-based visualization

The amino acid sequence of SARS-CoV-2 RBD (residue 319-521 of UniProtKB P0DTC2), with corresponding substitution mutations at G496S, Q498R, was submitted to the deep-learning based programme AlphaFold2 for structural prediction [17,18]. The first-ranking model of each mutant was selected and superimposed to RBD-ACE2 complex (PDB ID: 6M0J). RMSD of pruned atom pairs of each superimposed model: S496_RBD: 0.845Å; G496_R498__RBD: 0.740Å; S496_R498__RBD: 0.848Å.

### Generation of attenuated SARS-CoV-2 harbouring the spike mutations

An infectious molecular clone of SARS-CoV-2 on a BAC, named p-BAC-SARS-CoV-2, was generated and characterized as described previously [19]. The indicated point mutations were generated in p-BAC-SARS-CoV-2 using λ-Red-mediated homologous recombination. The primer sequences are listed in Supplementary Table 1. Validated BAC DNA was transfected into VeroE6-TMPRSS2 cells pre-seeded in a 6-well plate using Lipofectamine 3000 (Invitrogen) according to the manufacturer’s protocol. The cells were monitored for cytopathic effects on a daily basis. Virus-containing cells were harvested on 72 h post-infection (hpi). The titres of the viral stock were determined with a plaque assay using VeroE6 cells.

### Micro-neutralization assay

The SARS-CoV-2 micro-neutralization assay was performed as described previously [19]. Test serum samples were serially diluted, mixed with 50 μL of SARS-CoV-2 at 10^3^ PFU/ml in 96-well plates and incubated for 1 h at 37°C. The virus-serum mixtures were transferred to pre-seeded 96-well plates (VeroE6 cells, 2 × 10^4^ cells/well) and incubated for 24 h. After the incubation, the culture medium was removed, and the plates were air-dried in a biosafety cabinet (BSC) for 20 min. The cells were then fixed with 10% PBS-buffered formaldehyde for 30 min and air-dried in the BSC again. After permeabilization with 0.2% Triton X-100 in PBS, the cells were incubated with rabbit antiserum raised in-house against the SARS-CoV-2 N protein for 1 h at room temperature, followed by the addition of an Alexa Fluor 488-conjugated goat anti-rabbit IgG (H + L) cross-adsorbed secondary antibody (Life Technologies). SARS-CoV-2 foci were quantitated using a Sapphire Biomolecular Imager (Azure Biosystems).

### Statistical analysis

All data were analysed with Prism (GraphPad Software, Inc). *P* < 0.05 was considered statistically significant.

### Illustrations

The hamster illustrations and schematic figures were created with BioRender software (https://biorender.com/).

## Results

### The fitness advantage of BA.2 over BA.1

**Figure 1.**
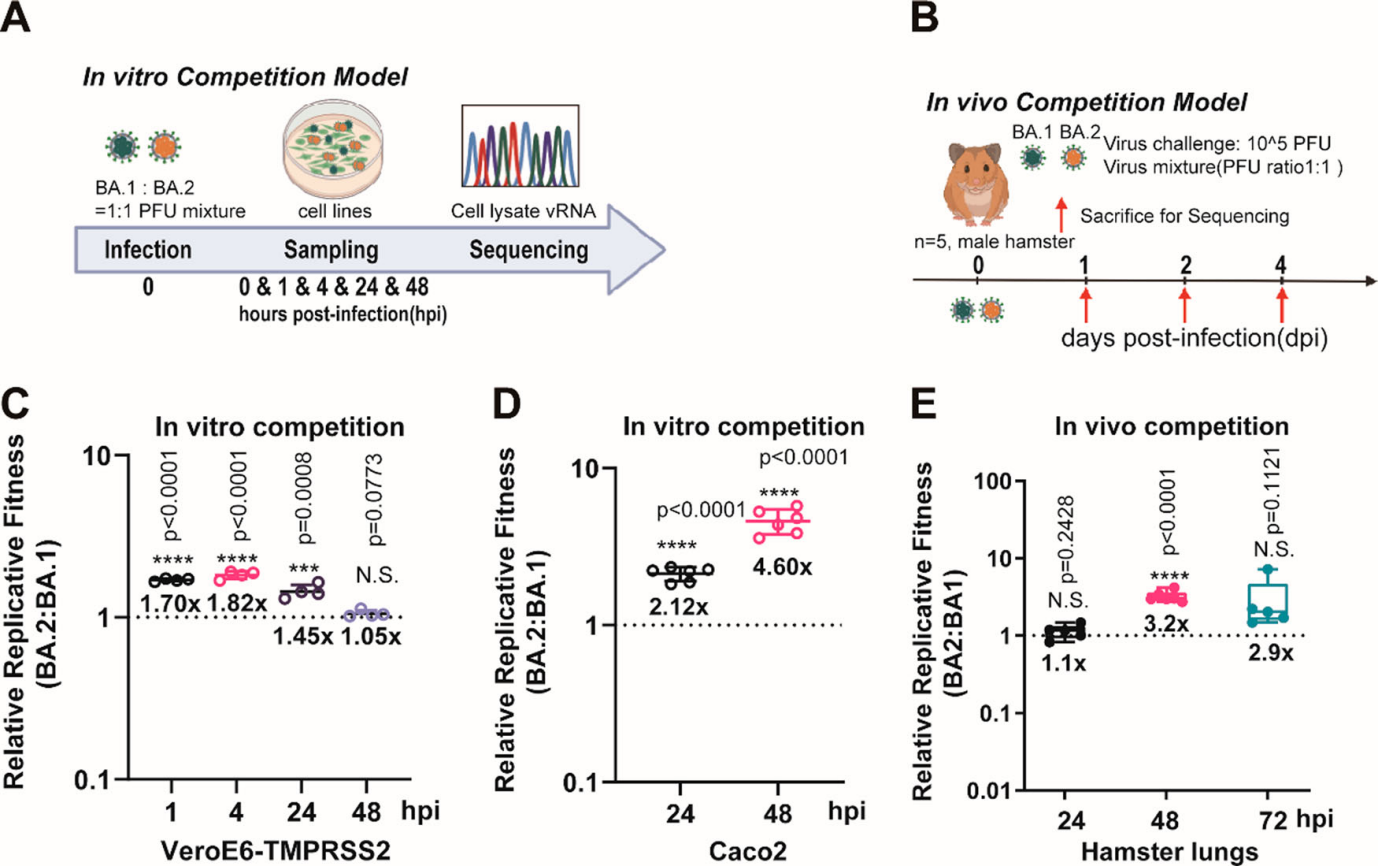
The replicative fitness advantage of Omicron BA.2 over BA.1. (A) Scheme of the *in vitro* competition model using a mixture of BA.1 and BA.2 variants (final MOI of 0.10 for each variant) in VeroE6-TMPRSS2 and Caco2 cell lines. (B) Scheme of the *in vivo* competition model using a mixture of both variants with an initial PFU ratio of 1:1 was inoculated in 8–10 weeks old male golden Syrian hamsters (*n* = 5 per group). At 0 dpi, each hamster was intranasally inoculated with 100 µL of DMEM containing 10^5^ PFU of each variant (*n* = 5 hamsters per time-point). (C) The BA.2 to BA.1 viral-load ratios in the VeroE6-TMPRSS2 cell lysates were measured by Sanger sequencing at indicated time points. (D) The BA.2 to BA.1 viral-load ratios in the Caco2 cell lysates were measured by Sanger sequencing at indicated time points. (E) The BA.2 to BA.1viral-load ratios of indicated time-point of hamster lung samples were determined by Sanger sequencing. Data in (C–E) are indicated as mean ± SD. *P* values are calculated as coefficient of each linear regression analysis of indicated time-point ratios versus baseline ratios (1:1). N.S., not significantly different; ***P < 0.001; ****P < 0.0001.

We first demonstrated that BA.1 and BA.2 exhibited similar replication kinetics in both upper and lower respiratory tracts of Syrian hamsters (Supplementary Figure 1). The peak viral titres in both nasal turbinate and lung tissues were detectable on as early as 2 days post-infection (dpi), and maintained until 4 dpi. On 7 dpi, the viral titres in both tissues were close to or below the detection limit (<100 TCID_50_/mL) in both groups of hamsters. We then compared the *in vitro* and *in vivo* fitness of BA.1 and BA.2 by mixing the two viruses in a PFU ratio of 1:1 (Figure 1A,B). Sanger sequencing validation was utilized to reliably quantify the relative amounts of the BA.1 and BA.2 variants in the mixed specimens (Supplementary Figure 2A). Our results indicated that BA.2 consistently exhibited higher fitness advantage over BA.1 at two cell culture models of VeroE6-TMPRSS2 and Caco2 cells (Figure 1C,D), as well as in hamster lungs (Figure 1E).

### The spike G496S substitution reduces replicative fitness of Omicron BA.1 against BA.2

**Figure 2.**
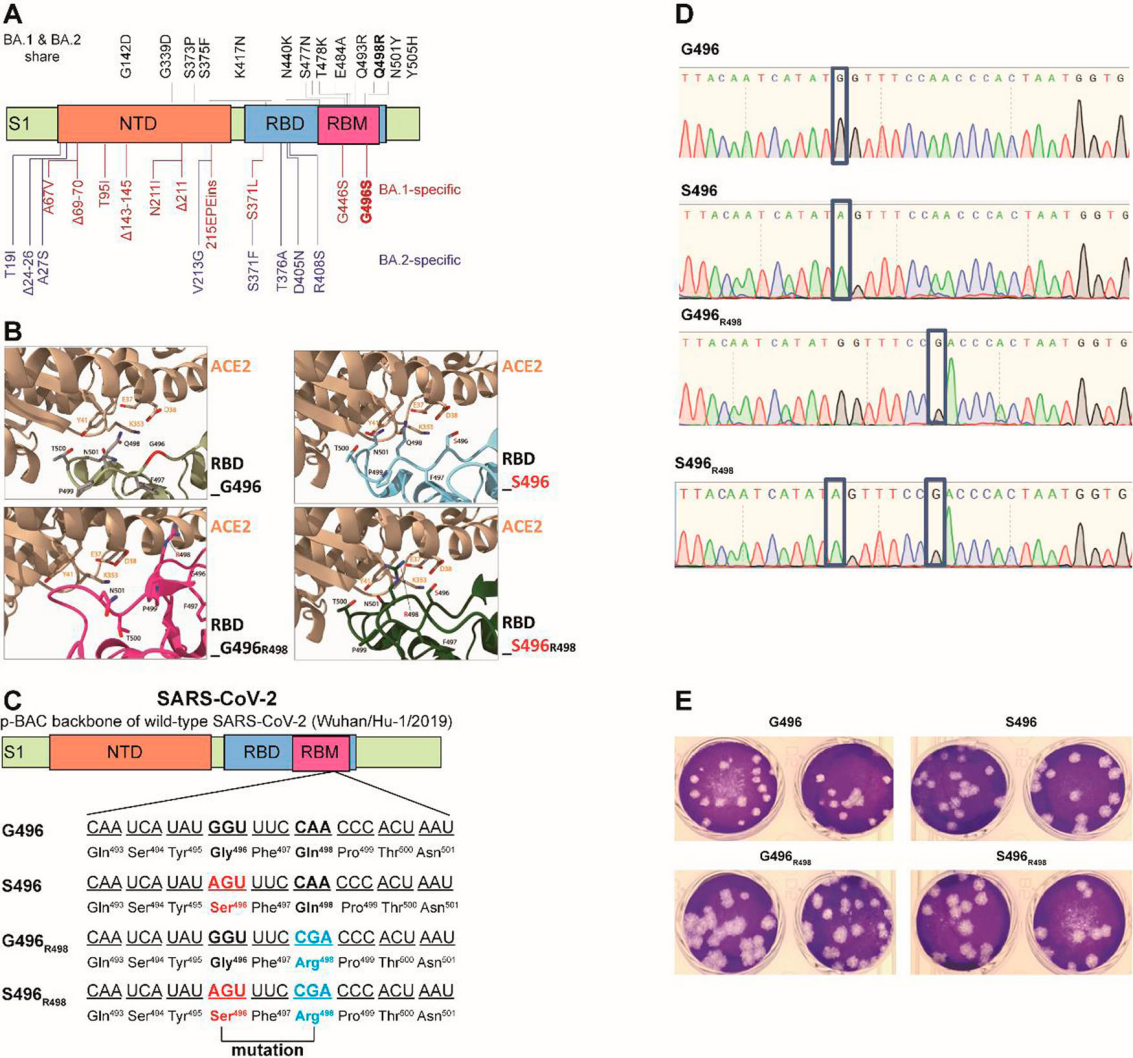
Construction of SARS-CoV-2 mutants and plaque morphologies of different recombinant mutants. (A) Schematic summary of mutations of BA.1 and BA.2 in the spike protein S1 domain. (B) The binding interface between human ACE2 (sand) and SARS-CoV-2 RBD wildtype (G496), S496, G496_R498,_ and S496_R498_ mutations. Key residues at the binding interface were labelled and shown in stick representation. (C) Construction of revertant SPIKE-G496S (S496), -Q498R (G496_R498_), and -double mutants (S496_R498_) SARS-CoV-2 based on the p-BAC backbone of wild-type SARS-CoV-2 (Wuhan/Hu-1/2019). (D) Sanger sequencing of wildtype-backbone (G496), S496, G496_R498,_ and S496_R498_ mutations. (E) Plaque morphologies of wildtype-backbone (G496), S496, G496_R498,_ and S496_R498_ mutations. The plaque images were taken on 60hpi and on VeroE6-TMPRSS2 cells.

**Figure 3.**
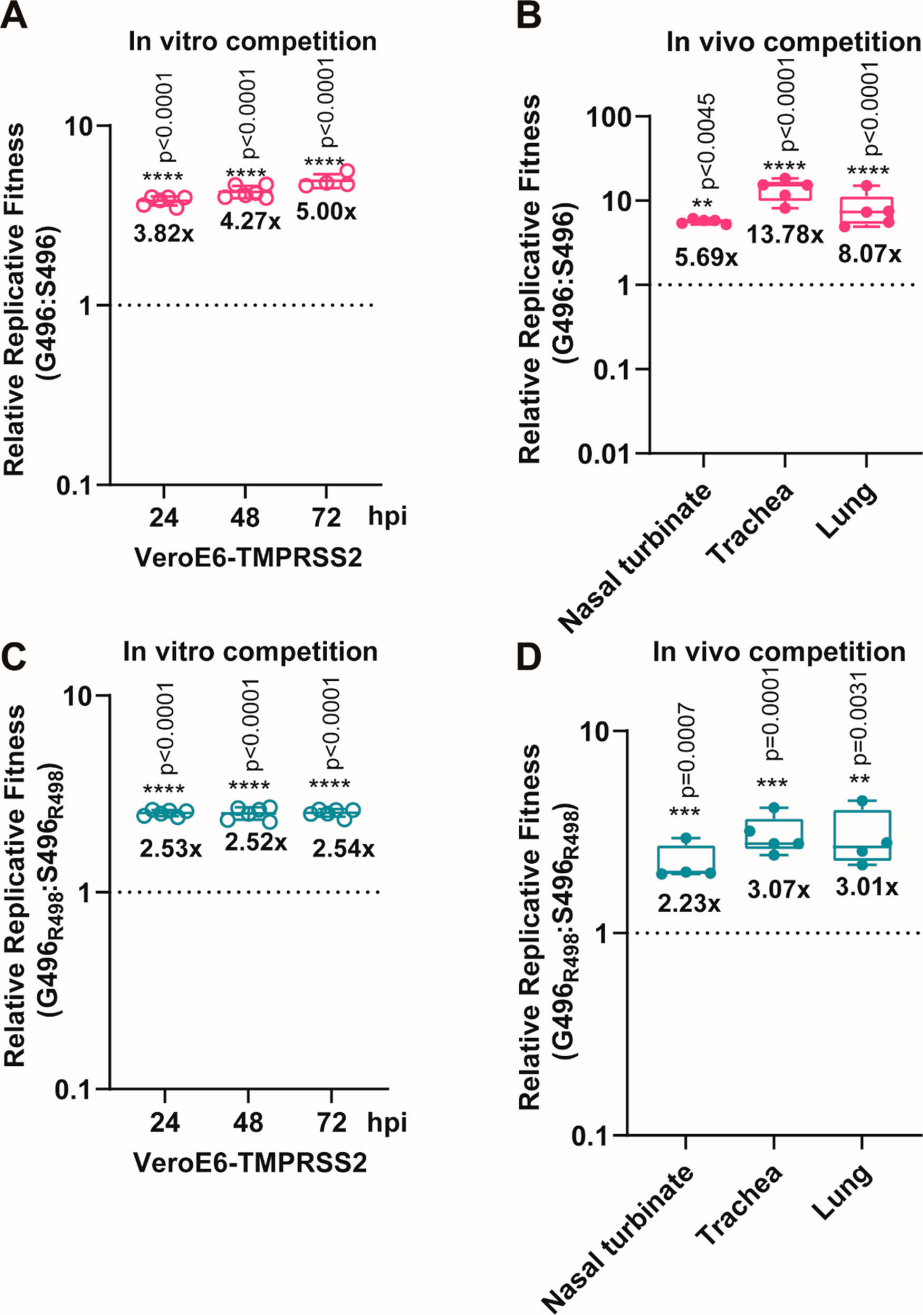
The spike G496S substitution reduces replicative fitness of Omicron BA.1 against BA.2 (A) *In vitro* virus competition assay using a mixture of the wildtype (G496) and S496 mutant. The viral-load ratios of G496 to S496 in the cell supernatants were measured by Sanger sequencing. (B) *In vivo* virus competition assay measuring the wildtype (G496) to S496 viral-load ratios in the nasal turbinate, trachea, and lung of male hamsters (*n* = 5 hamsters) at 2 dpi by Sanger sequencing. (C) *In vitro* virus competition assay using a mixture of the G496_R498_ single mutant and the S496_R498_ double mutant. The viral-load ratios of G496_R498_ to the S496_R498_ in the cell culture supernatants were determined by Sanger sequencing. (D) *In vivo* virus competition assay measuring the viral-load ratios of G496_R498_ single mutant to the S496_R498_ double mutant were performed in male hamsters (*n* = 5 hamsters) at 2 dpi by Sanger sequencing. All data are indicated as mean ± SD. *P* values are calculated as the coefficient of each linear regression analysis of indicated group ratios versus baseline ratios (1:1). **P < 0.01; ***P < 0.001; ****P < 0.0001.

Next, we explored the potential molecular determinant(s) that are responsible for the enhanced fitness of BA.2 over BA.1. At the spike RBD, BA.1 and BA.2 share 12 RBD mutations (ie: G339D, S373P, S375F, K417N, N440K, S477N, T478K, E484A, Q493R, Q498R, N501Y, and Y505H). BA.1 has the distinct RBD mutations S371L, G446S, and G496S, while BA.2 has S371F, T376A, D405N, and R408S (Figure 2A). Among these mutations, G446S and G496S are the only two found in the ACE2 receptor-binding motif (RBM). Given that G496 was predicted to stabilize RBD-ACE2 binding [20], we prioritized the G496S substitution for functional analysis in this study. To this end, we mapped the effects of G496S mutation onto the ACE2-bound SARS-CoV-2 RBD crystal structure and found that G496 had Van-der-Waal’s contact with D38 and K353 on ACE2, locating 6.3Å and 9.0Å away from the two residues, respectively (Figure 2B). We deduce that mutation of glycine, the smallest amino acid, to any larger residues including serine, may lead to unfavourable conformational changes in the protein interface, thereby reducing RBD-hACE2 binding. To validate this hypothesis, we attempted to generate recombinant virus using wild-type SARS-CoV-2 (Wuhan/Hu-1/2019) with single G496S mutation (Figure 2C). The resulting single G496S mutation developed equivalent plaque morphology compared with wild-type SARS-CoV-2 (Figure 2D,E). In our *in vitro* competition assay, the successfully-rescued mutant virus S496 had 4–5 folds lower fitness than wild-type SARS-CoV-2 in VeroE6-TMPRSS2 cells (Figure 3A). In line with this *in vitro* observation, 5–10 folds lower replicative fitness was found in S496-infected hamsters’ nasal turbinate, trachea, and lung tissues (Figure 3B). To further validate this phenomenon in the context of other Omicron mutations, we introduced Q498R which is a shared RBM mutation found in both BA.1 and BA.2. Of note, synergy between R498 and other Omicron RBD mutations has been reported to reinforce these mutations’ biological significance [21]. A double mutant (S496_R498_) recombinant virus carrying both S496 and R498 substitutions was generated using reverse genetics and validated by Sanger sequencing for competition studies (Supplementary Figure 2B,C). As shown in Figure 3C, the S496_R498_ double mutant virus consistently exhibited lower fitness up to 72 hpi in the VeroE6-TMPRSS2 cells. Similarly, 2–3 folds higher fitness was recorded in the hamster respiratory tract issues when G496S substitution was removed from the S496_R498_ double mutant virus (Figure 3D). Because G496S is found only in BA.1 but not BA.2, we concluded that the G496S substitution contributed to the lower fitness of BA.1 than BA.2.

### The spike G496S substitution recapitulates the immunoevasive phenotype of BA.1 and BA.2

**Figure 4.**
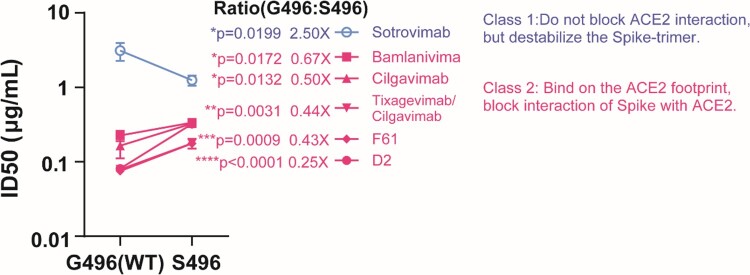
mAb neutralizing activity against S496 and G496 variants. Pairwise neutralizing antibody titres (half-maximum inhibitory dose; ID_50_) against S496 single mutant and G496 wildtype. The mAbs are divided into two classes according to their opposite phenotypes of antibody evasion. The result is shown as mean ± SD of triplicate and repeated twice for confirmation. Data is analysed by unpaired *t* test. *P < 0.05; **P < 0.01; ***P < 0.001; ****P < 0.0001.

BA.1 and BA.2 are antigenically equidistant from wild-type SARS-CoV-2 and thus similarly immunoevasive to antibody response elicited by existing COVID-19 vaccines [3]. Intriguingly, BA.1 and BA.2 exhibited noticeable differences in their sensitivity to therapeutic monoclonal antibodies [10]. Thus, we asked if G496S substitution differentiates the capacity of antibody evasion between BA.1 and BA.2, too. A panel of monoclonal antibodies were examined. Some are authorized therapies including Vir Biotechnology (Sotrovimab, VIR-783), Lilly (Bamlanivimab, LY-CoV555) and AstraZeneca (Tixagevimab/Cilgavimab). Neutralization activity of the selected mAbs against S496 mutant were compared with that of G496 (WT). Remarkably, neutralizing activity of class 2 mAbs, which bind on or in proximity to RBD to block Spike-ACE2 binding [22,23], decreased 15% to 50% against S496 mutant when compared with the G496 WT (Figure 4). In contrast, Sotrovimab that does not compete with ACE2 binding [24], exhibited generally higher neutralizing activity against S496 mutant than that of WT (Figure 4). In line with our observation, a recent work found that Sotrovimab lost neutralization activity against BA.2 but maintained against BA.1, whereas Cilgavimab exhibited better sensitivity against BA.2 than BA.1 [10]. Taken together, we concluded that the BA.1-specific G496S substitution contributed to the differentiated antibody resistance of Omicron sublineages.

## Discussion

In this manuscript, we comprehensively characterized the replication fitness of SARS-CoV-2 sublineage *in vitro* and *in vivo* and pinpointed G496S as an important substitution to affect both replication advantage and immunoevasive phenotype of BA.1. Our study has limitations. Ideally, distant mutations between BA.1 and BA.2 should be compared in the context of their mutually-shared backbone instead of the reference wild-type strain. For example, Q498R itself has been shown to have negative effects on hACE2 binding and stability in a deep-mutational scanning of single RBD mutations [25]. In an *in vitro* evolution study of SARS-CoV-2 variants, Q498R did not establish itself independently, but only emerged together with N501Y. Of note, the combination of two mutations has higher binding affinity to ACE2 compared to N501Y alone [26]. Like R498 + Y501, there might also be an epistatic effect between R498 and S496, in which S496 positions R498 to form multiple polar interactions at the RBD-ACE2 interface, including a hydrogen bond with Q42 of ACE2. Nevertheless, we pinpointed the dual role of the spike RBM substitution G496S in affecting virus SARS-CoV-2 fitness and properties of antibody evasion. Close surveillance of spike amino acid 496 position shall be performed to monitor this important determinant during the evolutionary trajectory of SARS-CoV-2.

## Supplementary Material

Supplemental Material
