# Supplementary material for: The spike receptor-binding motif G496S substitution determines the replication fitness of SARS-CoV-2 Omicron sublineage
Source: Emerg Microbes Infect. 2022 Aug 31;11(1):2093–101. doi: 10.1080/22221751.2022.2111977 (PMC9448440; doi:10.1080/22221751.2022.2111977)
Supplement: Supplemental Material [file TEMI_A_2111977_SM5760.pdf]

Supplementary Materials for

The spike receptor-binding motif G496S substitution determines the replication fitness of SARS-CoV-2 Omicron sublineage

The file includes:

Supplementary Table 1

Supplementary Figures 1 to 3

Supplementary Material

Supplementary Table 1

Primer	Sequence 5’ – 3’
galK-F‘	GCACACCTTGTAATGGTGTTGAAGGTTTTAATTGTTAC<br>TTTCCTTTACAACCTGTTGACAATTAATCATCGGCA
galK-R‘	GTAGAAGTTCAAAAGAAAGTACTACTACTCTGTATGG<br>TTGGTAACCAACATCAGCACTGTCCTGCTCCTT
S496-F’	GCACACCTTGTAATGGTGTTGAAGGTTTTAATTGTTAC<br>TTTCCTTTACAATCATATAGTTTCCAACCCACTAATGG<br>TGTTGGTTACCAACCATACAGAGTAGTAGTACTTTCTT<br>TTGAACTTCTAC
S496-R’	GTAGAAGTTCAAAAGAAAGTACTACTACTCTGTATGG<br>TTGGTAACCAACACCATTAGTGGGTTGGAAACTATAT<br>GATTGTAAAGGAAAGTAACAATTAAAACCTTCAACAC<br>CATTACAAGGTGTGC
G496$_{R498}$-F’	GCACACCTTGTAATGGTGTTGAAGGTTTTAATTGTTAC<br>TTTCCTTTACAATCATATGGTTTCCGACCCACTAATGG<br>TGTTGGTTACCAACCATACAGAGTAGTAGTACTTTCTT<br>TTGAACTTCTAC
G496$_{R498}$-R’	GTAGAAGTTCAAAAGAAAGTACTACTACTCTGTATGG<br>TTGGTAACCAACACCATTAGTGGGTCGGAAACCATAT<br>GATTGTAAAGGAAAGTAACAATTAAAACCTTCAACAC<br>CATTACAAGGTGTGC
S496$_{R498}$-F’	GCACACCTTGTAATGGTGTTGAAGGTTTTAATTGTTAC<br>TTTCCTTTACAATCATATAGTTTCCGACCCACTAATGG<br>TGTTGGTTACCAACCATACAGAGTAGTAGTACTTTCTT<br>TTGAACTTCTAC
S496$_{R498}$-R’	CCCACTAATGGTGTTGGTTACCAACCATACAGAGTAG<br>TAGTACTTTCTTTTGAACTTCTACTCGGAAACT<br>ATATGATTGTAAAGGAAAGTAACAATTAAAACCTTCA<br>ACACCATTACAAGGTGTGC

* Targeted amino acid sequences are underlined.

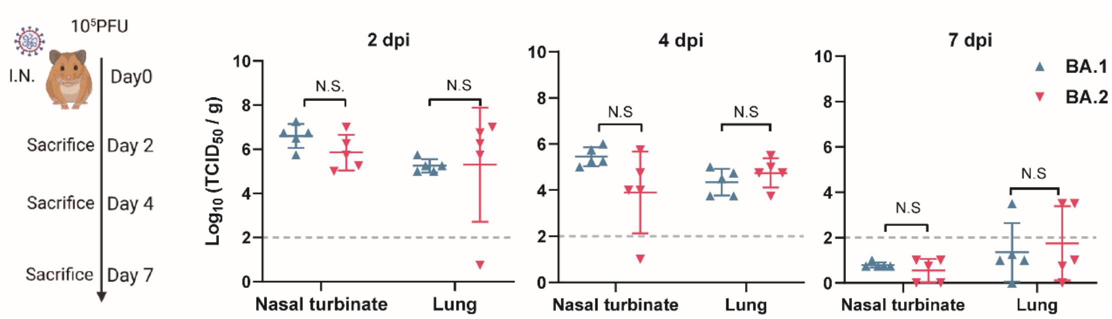

Supplementary Figure 1. Replication kinetics of BA.1 and BA.2 in hamsters. Nasal turbinate and lung tissues were serially collected from the Omicron BA.1-infected and Omicron BA.2-infected male Syrian hamsters (n = 5 per group) at the indicated time-points for virus titration by $TCID_{50}$ assays. Data represent mean ± standard deviations. N.S., not significantly different by Student's t-test.

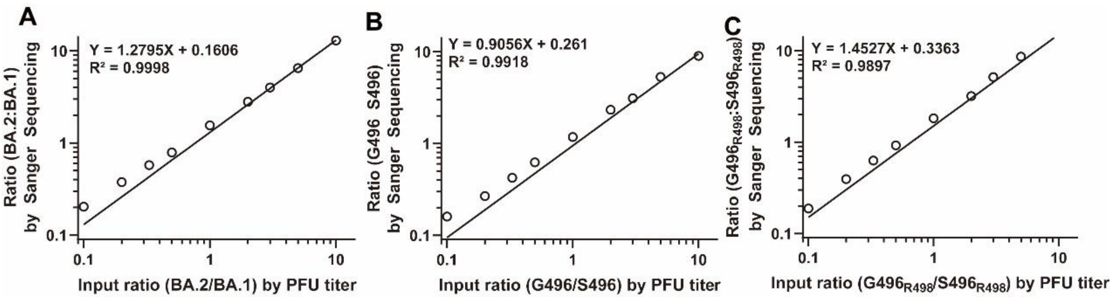

Supplementary Figure 2. Validation of the competition assay. The correlation between input PFU ratios and output RT-PCR amplicon ratios determined by Sanger sequencing. The BA.1 and BA.2 variants were mixed at PFU ratios of 10:1, 5:1, 3:1, 1:1, 1:3, 1:5, or 1:10. Total RNA of the mixture was extracted and amplified by RT-PCR. Ratio was calculated by the peak heights of Sanger sequencing. **(A)** Ratios of BA.2 to BA.1. **(B)** Ratios of G496 mutant virus to S496 virus. **(C)** Ratios of $G496_{R498}$ single mutant virus to $S496_{R498}$ double mutant virus.

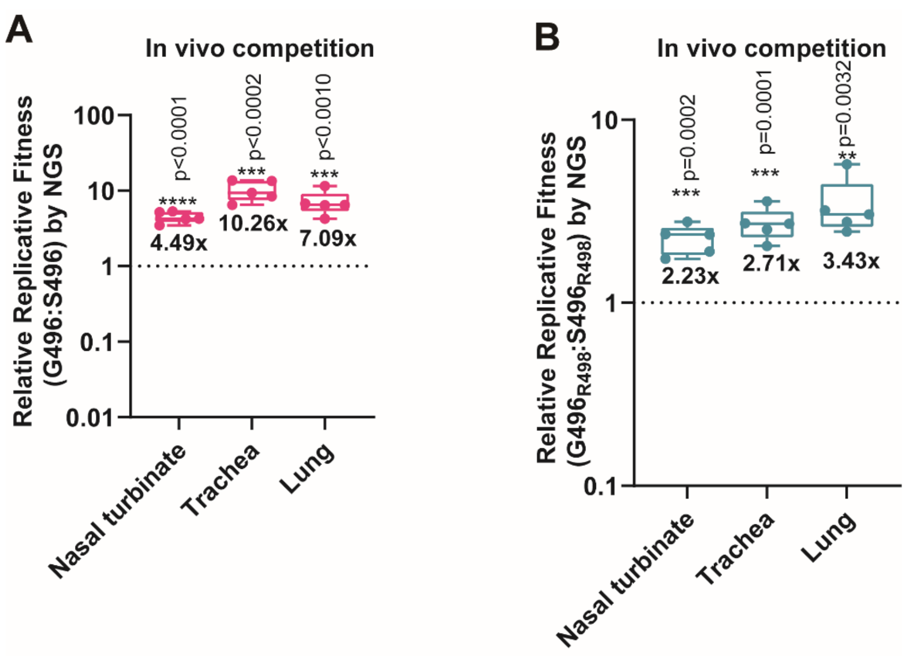

Supplementary Figure 3. (A) *In vivo* virus competition assay measuring the wildtype (G496) to S496 viral-load ratios in the nasal turbinate, trachea, and lung of male hamsters (n=5 hamsters) at 2 days post-infection (2dpi) by NGS. **(B)** *In vivo* virus competition assay measuring the viral-load ratios of $G496_{R498}$ single mutant to the $S496_{R498}$ double mutant were performed in male hamsters (n=5 hamsters) at 2dpi by NGS. All data are indicated as mean ± SD. The ratios between the comparative variants were calculated based on the ratio of reads containing the 2 spike mutations, G496S and G498R. P values are calculated for the group (strain) coefficient for each linear regression analysis of indicated sample ratios versus baseline ratio (1:1). $^{**}P < 0.01$; $^{***}P < 0.001$; $^{****}P < 0.0001$.
